# Gamification of Cognitive Assessment and Cognitive Training: A Systematic Review of Applications and Efficacy

**DOI:** 10.2196/games.5888

**Published:** 2016-07-15

**Authors:** Jim Lumsden, Elizabeth A Edwards, Natalia S Lawrence, David Coyle, Marcus R Munafò

**Affiliations:** ^1^ UK Centre for Tobacco and Alcohol Studies School of Experimental Psychology University of Bristol Bristol United Kingdom; ^2^ MRC Integrative Epidemiology Unit (IEU) University of Bristol Bristol United Kingdom; ^3^ Centre for Primary Care and Public Health Bart's & The London School of Medicine & Dentistry London United Kingdom; ^4^ Department of Psychology College of Life and Environmental Sciences University of Exeter Exeter United Kingdom; ^5^ School of Computer Science University College Dublin Dublin Ireland

**Keywords:** gamification, gamelike, cognition, computer games, review

## Abstract

**Background:**

Cognitive tasks are typically viewed as effortful, frustrating, and repetitive, which often leads to participant disengagement. This, in turn, may negatively impact data quality and/or reduce intervention effects. However, gamification may provide a possible solution. If game design features can be incorporated into cognitive tasks without undermining their scientific value, then data quality, intervention effects, and participant engagement may be improved.

**Objectives:**

This systematic review aims to explore and evaluate the ways in which gamification has already been used for cognitive training and assessment purposes. We hope to answer 3 questions: (1) Why have researchers opted to use gamification? (2) What domains has gamification been applied in? (3) How successful has gamification been in cognitive research thus far?

**Methods:**

We systematically searched several Web-based databases, searching the titles, abstracts, and keywords of database entries using the search strategy (gamif* OR game OR games) AND (cognit* OR engag* OR behavi* OR health* OR attention OR motiv*). Searches included papers published in English between January 2007 and October 2015.

**Results:**

Our review identified 33 relevant studies, covering 31 gamified cognitive tasks used across a range of disorders and cognitive domains. We identified 7 reasons for researchers opting to gamify their cognitive training and testing. We found that working memory and general executive functions were common targets for both gamified assessment and training. Gamified tests were typically validated successfully, although mixed-domain measurement was a problem. Gamified training appears to be highly engaging and does boost participant motivation, but mixed effects of gamification on task performance were reported.

**Conclusions:**

Heterogeneous study designs and typically small sample sizes highlight the need for further research in both gamified training and testing. Nevertheless, careful application of gamification can provide a way to develop engaging and yet scientifically valid cognitive assessments, and it is likely worthwhile to continue to develop gamified cognitive tasks in the future.

## Introduction

Every day, millions of people play games, on computers, consoles, and mobile devices [1]. One explanation for the massive popularity of games is that they can provide easy access to a sense of engagement and self-efficacy which reality may not deliver [2]. By their nature, games present users with difficult challenges to overcome and use narrative structure, complex graphics, strategic elements, and intuitive rules to engage the user [3]. This ability to engross users has recently begun to be leveraged for purposes beyond entertainment in the form of gamification. The aim of gamification is to use gamelike features (competition, narrative, leaderboards, graphics, and other game design elements) to transform an otherwise mundane task into something engaging and even fun.

In cognitive science, whether we are gathering data or trying to encourage behavior change, successfully engaging participants is vital. There is evidence that a lack of participant motivation has a negative impact on the quality of data collected [4], with tasks commonly being viewed as too boring and repetitive for participant attention to be sustained. This is a particular problem for Web-based studies where participants can simply close the browser window and drop out of the study the moment they decide it is not worth their time [5].

If we are attempting to alter behavior (for example, cognitive bias modification or working memory training), then it is vital that our interventions are engaging. Gamification may help in this regard. If we can successfully import game design elements into cognitive tasks without undermining their scientific value, then we may be able to improve the quality of our data, increase the effectiveness of our interventions, and improve the experience for participants. Furthermore, using games as a vehicle to deliver cognitive training may also be advantageous simply because video games appear to have positive effects on a number of outcomes, including working memory, attentional capacity, problem solving, motivation, emotional control, and prosocial behaviors [6]. In essence, delivering targeted cognitive training through a video game medium might provide a range of benefits.

Much of the literature on gamification is relatively recent, cross-disciplinary, and heterogeneous in nature. This lack of coherence in the field is partly due to poor definition of terms; for example, the gamelike tasks covered in this review could be described as “serious games,” “gamelike,” “gamified,” “games with a purpose,” “gamed-up,” or simply “computer based” [7-10]. To our knowledge, there have been no systematic reviews of gamification in cognitive research. There have, however, been several reviews of the concepts core to gamification and how they can be applied successfully (see [11-13]). However, detailed discussion of these reviews is beyond the scope of this paper. Also related but beyond our scope is the body of work on the effects of video games on behavior (see [14,15] for reviews).

This paper aims to systematically review the ways in which gamification has already been used for the purposes of cognitive assessment and training. We were specifically interested in the following questions: (1) Why have researchers opted to use gamification? (2) What domains has gamification been applied in? (3) How successful has gamification been in cognitive research thus far? We deliberately used a broad search strategy so as not to miss any relevant papers. We reviewed only the peer-reviewed literature and have therefore deliberately excluded some cognitive training games available on the iTunes or Play Store (such as Luminosity and Peak) unless supported by peer-reviewed research.

## Methods

### Databases and Search Strategy

The following databases were searched electronically: PsycInfo, Medline, ETHOS, EMBASE, PubMed, IBSS, Francis, Web of Science and Scopus. We searched the titles, abstracts, and keywords of database entries using the search strategy (gamif* OR game OR games) AND (cognit* OR engag* OR behavi* OR health* OR attention OR motiv*), where * represents a wildcard to allow for alternative suffixes. Searches included papers published in English between January 2007 and October 2015. We searched the bibliographies of included papers to locate further relevant material not discovered in the database search.

### Inclusion Criteria

#### Primary Research Paper

Included papers were empirical research studies, not literature reviews, opinion pieces, or design documents.

#### Novel Gamelike Task

Included papers focussed on newly developed gamelike tasks, created specifically for the study in question. We excluded commercially available video games (ie, “off-the-shelf” games) as well as gamelike tasks that have been in use for many years, such as Space Fortress (see [16] for a review).

#### Measure or Train Cognition

Included papers focussed on tasks designed to assess or train cognition. For scoping purposes, we took a narrow definition of cognition: those processes involved in memory, attention, decision making, impulse control, executive functioning, processing speed, and visual perception.

#### Validated or Piloted

Included papers had to involve an empirical study, either validating the task as a measure of cognition or piloting the intervention. Papers regarding usability testing alone were excluded.

### Exclusion Criteria

#### Non–Peer-Reviewed Papers

We excluded non–peer-reviewed papers such as abstracts or conference posters.

#### Gamification in the Behavioral Sciences but not Involving Cognition

We excluded papers on gamification for education purposes, disease management, health promotion, exposure therapy, or rehabilitation.

#### Game Engines/Three-Dimensional (3D) Environments

We excluded papers that made use of virtual reality or a 3D environment without any game mechanics or gamelike framing.

### Screening

We did not select papers based on whether they included “gamification.” Rather, papers were selected if they were captured by our search strategy and were considered relevant based on our inclusion/exclusion criteria. We intentionally did not strictly define gamification: as has been discussed famously by Wittgenstein and is alluded to by Deterding [13], trying to precisely define what elements make a game is both difficult and limiting. Therefore, we decided that a task was gamified if its stated purpose was to increase participant motivation. Where there was insufficient detail to determine whether a paper met our inclusion criteria, we erred on the side of caution to increase our confidence in the relevance of the studies reviewed.

After screening, data were extracted from each paper using a standardized data extraction form. Data relating to the questions of interest such as application of gamification, approach taken, and efficacy were extracted from each paper. Application of gamification refers to the field of psychology in which the gamelike task was used and why a gamelike task was used. Approach taken refers to the specific game mechanics used in the task and what themes and scaffolding were applied. Finally, efficacy refers to the findings produced by the task in practice, as well as details on the participants, methods used for evaluation, and limitations of the study. Categorization of concepts (such as the cognitive domains measured) was done using the paper-authors’ own words where possible. Where not possible, we mapped extracted concepts closely to existing categories.

All papers identified by our search strategy were screened by one reviewer (JL) in 3 stages, to determine whether they were relevant based on our inclusion/exclusion criteria: title, abstract, and full text. A second reviewer (EE) rescreened 20% of the papers from the title stage onward to ensure that no relevant papers were missed. Papers were only included in the review on the agreement of both JL and EE.

## Results

### Search Results

**Figure 1:**
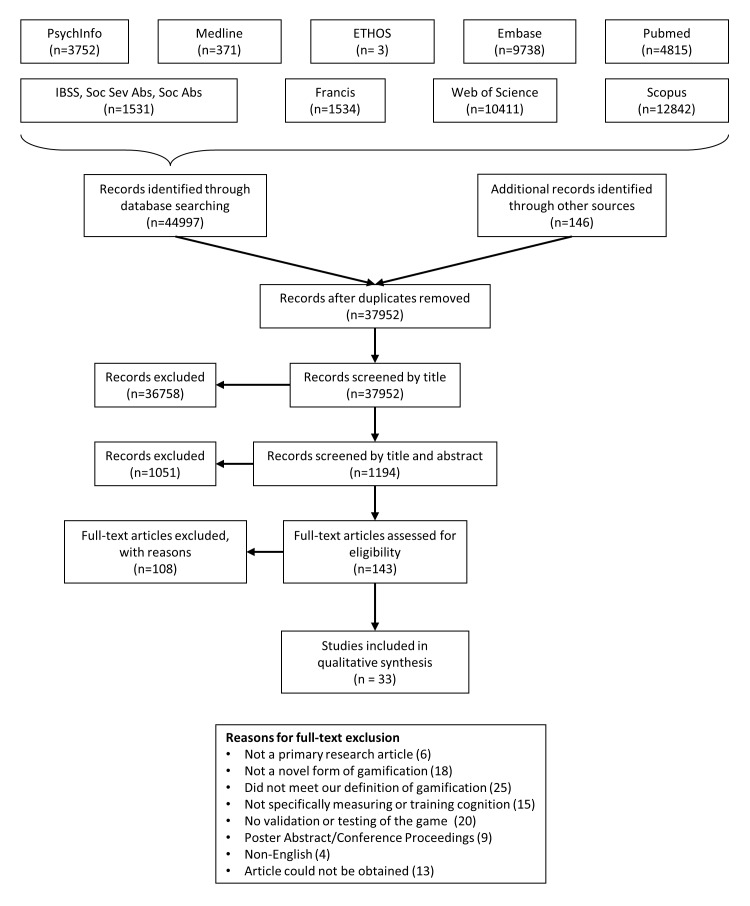
Flowchart detailing the paper discovery and screening process.

Our initial search yielded 33,445 papers (excluding duplicates). Of these, 23 papers from the original search and 4 papers from the manual reference search were included in the review. We repeated the search in October 2015, including papers from January 2015 until October 2015. This search produced 4448 papers (excluding duplicates) and resulted in another 4 papers being included in the review, with a further 2 also included following peer review. The total number of papers included in the review was therefore 33. See Figure 1 for a flowchart of the combined searches and Table 1 for details of all included studies. We used Cohen *K* to assess inter-rater reliability of paper inclusion at the 20% data check stage (7590 papers checked). There was moderate agreement between the 2 reviewers (*k*=.526, 95% CI, 0.416 to 0.633, *P*<.001). All supplementary data referenced in this paper can be found in Multimedia Appendix 1, whereas Multimedia Appendix 2 contains more detailed information on the games and game mechanics used by studies in the review.

**Table 1:** Details of included papers.^a^

Author, year	Full title	Game	Category
McPherson and Burns, 2007 [17]	Gs invaders: Assessing a computer gamelike test of processing speed	Space Code	Testing
McPherson and Burns, 2008 [18]	Assessing the validity of computer gamelike tests of processing speed and working memory	Space Matrix/Space Code	Testing
Trapp et al, 2008 [19]	Cognitive remediation improves cognition and good cognitive performance increases time to relapse—results of a 5-year catamnestic study in schizophrenia patients	Xcog	Training
Gamberini et al, 2009 [20]	Eldergames project: An innovative mixed reality table-top solution to preserve cognitive functions in elderly people	Eldergames	Testing
Gamberini, Cardullo, Seraglia, and Bordin, 2010 [21]	Neuropsychological testing through a Nintendo Wii console	Wii Tests	Testing
Dovis, Oord, Wiers, and Prins, 2011 [22]	Can motivation normalize working memory and task persistence in children with attention-deficit/hyperactivity disorder? The effects of money and computer-gaming	Megabot	Testing
Delisle and Braun, 2011 [23]	A context for normalizing impulsiveness at work for adults with attention deficit/hyperactivity disorder (combined type)	Retirement Party	Testing
Prins, Dovis, Ponsioen, ten Brink, and van der Oord, 2011 [24]	Does computerized working memory training with game elements enhance motivation and training efficacy in children with ADHD?	Supermecha	Training
Lim et al, 2012 [25]	A brain-computer interface based attention training program for treating attention deficit hyperactivity disorder	Cogoland	Training
Heller et al, 2013 [26]	A machine learning-based analysis of game data for attention deficit hyperactivity disorder assessment	Groundskeeper	Testing
Hawkins et al, 2013 [9]	Gamelike features might not improve data	EM-Ants and Ghost Trap	Testing
Verhaegh, Fontijn, Aarts, and Resing, 2013 [27]	In-game assessment and training of nonverbal cognitive skills using TagTiles	Tap the Hedgehog	Both
Aalbers, Baars, Rikkert, and Kessels, 2013 [28]	Puzzling with online games (BAM-COG): reliability, validity, and feasibility of an online self-monitor for cognitive performance in aging adults	BAM-COG	Testing
Fagundo et al, 2013 [29]	Video game therapy for emotional regulation and impulsivity control in a series of treated cases with bulimia nervosa	Playmancer	Training
Anguera et al, 2013 [30]	Video game training enhances cognitive control in older adults	Neuroracer	Training
van der Oord, Ponsioen, Geurts, Ten Brink, and Prins, 2014 [31]	A pilot study of the efficacy of a computerized executive functioning remediation training with game elements for children with ADHD in an outpatient setting	Braingame Brian	Training
Brown et al, 2014 [32]	Crowdsourcing for cognitive science—the utility of smartphones	The Great Brain Experiment	Testing
Tong and Chignell, 2014 [33]	Developing a serious game for cognitive assessment: choosing settings and measuring performance	Whack-a-mole	Testing
Katz, Jaeggi, Buschkuehl, Stegman, and Shah, 2014 [34]	Differential effect of motivational features on training improvements in school-based cognitive training	WMTrainer	Training
Dunbar et al, 2013 [8]	Implicit and explicit training in the mitigation of cognitive bias through the use of a serious game	MACBETH	Training
Lee et al, 2013 [35]	A brain-computer interface based cognitive training system for healthy elderly: A randomised control pilot study for usability and preliminary efficacy	Card-Pairing	Training
Miranda and Palmer, 2013 [36]	Intrinsic motivation and attentional capture from gamelike features in a visual search task	Visual Search	Testing
Atkins et al, 2014 [37]	Measuring working memory is all fun and games: A four-dimensional spatial game predicts cognitive task performance	Shapebuilder	Testing
Dörrenbächer et al, 2014 [38]	Dissociable effects of game elements on motivation and cognition in a task switching training in middle childhood	Watermons	Training
McNab and Dolan, 2014 [39]	Dissociating distractor-filtering at encoding and during maintenance	The Great Brain Experiment	Testing
O’Toole and Dennis, 2014 [40]	Mental health on the go: Effects of a gamified attention-bias modification mobile application in trait-anxious adults	ABMTApp	Training
Tenorio Delgado, Arango Uribe, Aparicio Alonso, and Rosas Diaz, 2014 [41]	TENI: A comprehensive battery for cognitive assessment based on games and technology	TENI	Testing
De Vries, Prins, Schmand, and Geurts, 2015 [42]	Working memory and cognitive flexibility-training for children with an autism spectrum disorder: a randomized controlled trial	Braingame Brian	Training
Dovis, Van Der Oord, Wiers, and Prins, 2015 [43]	Improving executive functioning in children with ADHD: Training multiple executive functions within the context of a computer game. A randomized double-blind placebo controlled trial	Braingame Brian	Training
Kim et al, 2015 [44]	Effects of a serious game training on cognitive functions in older adults	Smart Harmony	Training
Manera et al, 2015 [45]	“Kitchen and cooking,” a serious game for mild cognitive impairment and Alzheimer’s disease: a pilot study	Kitchen and Cooking	Both
Ninaus et al, 2015 [46]	Game elements improve performance in a working memory training task	GAME	Training
Tarnanas et al, 2015 [47]	On the comparison of a novel serious game and electroencephalography biomarkers for early dementia screening	VAP-M	Testing

^a^In cases where the game was not named, we assigned a descriptive name.

### Why Do Researchers Use Gamification?

We searched each paper for explanations as to why a gamelike task had been used and identified reasons for researchers opting to gamify their cognitive training and testing. These reasons were then coalesced into 7 categories. Some authors listed multiple reasons for gamifying their approach, whereas others gave no motivations at all. Supplementary Table 1 in Multimedia Appendix 1 provides details of which games fell into each category.

#### To Increase Participant Motivation

Although we assume that increasing participant motivation was a goal for every study in this review, we found 16 studies that explicitly used gamified tasks to measure or train cognition in a more motivating manner, and the majority (10 of 16) of these studies were assessment studies. Cognitive tests are typically a one off measure, and replayability is not a requirement. As such, these games tended to build simple game-archetypes, such as space invaders or whack-a-mole, around an existing cognitive task; with the goal of encouraging self-motivation, improving participant enjoyment, and even reducing test anxiety [17]. Gamification aims to increase engagement with tasks that might otherwise be perceived as demotivating [48], and the highly repetitive nature of cognitive tasks means they are ripe for improvement.

#### To Increase Usability/Intuitiveness for the Target Age Group

Eleven tasks were gamified specifically to enhance appeal with a given age group (see Supplementary Table 2 in Multimedia Appendix 1). The authors of these studies hypothesized that a more intuitive interface could prevent boredom and anxiety in the target age group, which might damage motivation and concentration on the tasks at hand. Six games were designed to be suitable for the elderly, who may not be familiar working with a mouse and keyboard [49]. Five other games were aimed at young children, and reframed the cognitive assessment as a game, to test the children under optimal mental conditions [41].

#### To Increase Long-Term Engagement

Commercial gamification is often used to create long-term interest around a user experience, product, or event [13]. Similarly, we found 11 studies (8 games) that used gamification to reduce participant dropout rates over a protracted testing or training programme. Many of these games were used in an unobserved, nonlaboratory setting; therefore, the tasks made use of motivational features that made the training and testing intrinsically more appealing and less of a burden to perform on a regular basis. A common feature was that task sessions were kept as short as possible to make them more convenient and to increase likelihood of completion [28,32]. For example, *The Great Brain Experiment* aimed to keep task sessions below 5 minutes in duration and found that the shortest task, the Stop Signal Task, was the most popular mini game.

#### To Investigate the Effects of Gamelike Tasks

Obviously, many of the studies in this review investigated the effects of gamelike tasks, however, only 5 studies explicitly stated that their aim was to assess the motivational and cognitive effects of gamelike features. Three of the 6 games were very simplistic, with only a few specific game mechanics and carefully designed nongame controls, to make the effects of the game mechanics on the data as apparent as possible. One game, *Watermons* [38], was designed, using Self Determination Theory [50,51], to maximize participant intrinsic motivation and make any motivational effects of game mechanics as apparent as possible.

#### To Stimulate the Brain

Six studies cited evidence that playing video games can be cognitively beneficial and/or stimulating as a key factor in their decision to gamify. The past decade has seen rapid growth in the investigation of the cognitive effects of video gaming, and findings have typically been positive. There is good evidence that video game players outperform nongamers on tests of working memory [52] visual attention [53,54] and processing speed [55,56]. The “active ingredient” of this cognitive enhancement effect is not yet known, but it comes as no surprise that researchers who are interested in training cognition are keen to include gamelike features in their training tasks.

#### To Increase Ecological Validity

Cognitive training has often suffered from a lack of transferability [57-59]. Although participants may improve at the training task, these improvements do not generalize to the real world. In a similar vein, cognitive assessment has been accused of being ecologically invalid [60]. A potential solution is to make tasks more realistic, and these tasks inevitably become gamelike as 3D graphics, sounds, and narrative are added. We identified 6 studies that used gamified tasks to test cognition in an engaging close-to-life environment or enhance transferability of learned skills. As Dunbar et al explain [8], games are uniquely suited to some forms of cognitive training as they give the player freedom to make choices and experience feedback on the effects of those choices; in other words, they provide opportunities for experiential learning [61].

#### To Increase Suitability for the Target Disorder

Gamified tasks may also be more appealing to patients with certain clinical conditions. Specifically, we found 6 studies (covering 4 games) designed for people with attention deficit/hyperactivity disorder (ADHD). It is commonly reported that ADHD patients are compulsive computer game players [23]. Furthermore, patients with ADHD react differently from controls to rewards and feedback: they prefer strong reinforcement and immediate feedback, as well as clear goals and objectives, all of which can easily be delivered in a gamelike environment [22,24,62].

### What Are the Application Areas of Gamification?

We found comparable numbers of games used for cognitive testing (17) and training (13), with one game that can be used for both testing and training (see Supplementary Table 3 in Multimedia Appendix 1). It is also worth noting that the numbers of studies investigating cognitive testing (17) and training (15) were very similar, and the slightly smaller proportion of games used to deliver cognitive training is likely explained by the relative recency of the field.

Supplementary Table 4 in Multimedia Appendix 1 shows the cognitive domains assessed. Working memory was the most commonly tested domain. This is likely due to its ease of testing and also because working memory deficit is a common symptom in many disorders. General executive function (EF), attention, and inhibition were also commonly tested domains. Many games tested several cognitive domains and/or general EF, highlighting the difficulty of examining gamification effects on specific cognitive domains (and therefore comparing performance to standard cognitive tasks). Supplementary Table 5 in Multimedia Appendix 1 shows a breakdown of training tasks by the cognitive domains they addressed. Again, working memory was a popular target, closely followed by EF and inhibitory control training. There is a smaller overlap in domains covered by cognitive training tasks; test batteries tend to test a wide range of domains, whereas training focuses on 1 or 2 domains exclusively.

### Does Gamification Work?

The studies we reviewed were generally enthusiastic about their use of gamified tasks, although given the diversity of study aims, this does not mean that all games worked as expected. Where reported, subjective and objective measures of participant engagement were positive. All studies that measured intrinsic motivation reported that the use of gamelike tasks improved motivation, compared with nongamified versions. We identified 21 of 33 studies that compared a gamelike task directly against a nongamified counterpart, and these studies can shed light on the specific effects of gamification on testing and training tasks.

Most gamified assessments were validated successfully. *Wii Tests* [21], *Shapebuilder* [37], *The Great Brain Experiment* [32,39], *BAM-COG* [28], and *Tap the Hedgehog* [27] were all found to produce output measures/scores that correlated fairly well with their non–gamelike counterparts, though mixed-domain measures were an issue (see Supplementary Table 1 in Multimedia Appendix 2 for full details of all games). Validation studies varied in their design, and some studies reported complex correlations between gamified and nongamified tasks with multiple outputs. However, sample correlations from some of the simpler validation studies suggest intertask correlations of 0.45-0.60 [28,37,39,63]. Broadly speaking, these were well-designed and well-powered studies, and together, they provide encouraging evidence that cognitive tests can be gamified and still be useful as a research tool.

Some studies found that their gamified tests were correlated with measures of multiple cognitive domains, in other words, they were mixed-domain measures. For example, use of exploratory factor analysis showed that *Whack-a-Mole’s* primary output measure was correlated with 2 of the 3 EFs of interest: inhibition (*r* (22)=.60, *P*<.001) and updating (*r* (22) = .35, *P*<.05) [33]. *Space Code* [17,18] had similar problems. The initial study was successful, with *Space Code’s* output measure correlating well with a conventional measure of processing speed (*r* (58)=.55, *P*<.001). However, a second paper detailing 2 experiments which aimed to replicate the previous finding found that *Space Code’s* correlations with measures of working memory, visuospatial ability, and processing speed were not stable [18]. The fact that *Space Code* was thought to be a fairly pure measure in one study and then was shown to be mixed-domain in the next highlights the fact that designing gamified cognitive tasks is difficult, and multiple, well-powered validation studies may be required to ensure a task is measuring what is intended.

Gamification also has the potential to invalidate a task. For example, *Retirement Party* was compared against the Continuous Performance Task-II in healthy controls and adults with ADHD. The Continuous Performance Task-II detected more commission errors from the ADHD adults as expected (mean [M]=56, standard deviation [SD]=13 vs M=46, SD=10), but *Retirement Party* did not (M=14.4, SD=5.8 vs M=13.2, SD=4.3): this likely invalidates the game as a diagnostic tool for ADHD. However, Delisle and Braun [23] discuss the possibility that *Retirement Party* may have detected no deficit in ADHD patients as the nature of the task was such that there was no deficit: the highly structured and feedback-rich multitask environment may have normalized the ADHD patients’ usual inattention. Such a performance boost resulting from gamelike elements is a disadvantage when performing cognitive assessment but is likely to be desirable in a cognitive training scenario.

Several studies in this review focused specifically on adding game mechanics to cognitive tasks to investigate the resultant changes in data, enjoyment, and motivation. Dovis et al [22] studied whether different types of incentive could normalize ADHD children’s performance on working memory training. They found that regardless of incentive, ADHD children did not perform as well as healthy controls. However, ADHD children also experienced a decrease in performance over time, and the €10 condition and the gaming condition (*Megabot*) prevented this decrease. These results indicate that performance problems in ADHD training might be somewhat alleviated through the use of gamelike tasks. This is further supported by the study by Prins et al (*Supermecha* [24]), which found that ADHD children completed more training trials (M=199.48, SD=47.46 vs M=134.43, SD=34.18), with higher accuracy (69% vs 51%), when trained using a gamified working memory training task as opposed to a non–gamelike one. Children in the gamelike condition were also more engaged and enjoyed the training more, as measured by “absence time.” In a similar vein, The Great Brain Experiment [39] and GAME [46] both showed gamelike tasks to be appropriate for measuring and training working memory. With Ninaus et al, presenting evidence that gamification can improve overall participant performance in a working memory training task [48] and McNab and Dolan showing that data collected from 2 very different gamified and nongamified tasks could fit similar models of working memory capacity.

In contrast, *WMTrainer*, was assessed across 7 different conditions containing different combinations of game mechanics [34]. They compared training improvement across conditions and found that the greatest training effect was caused by versions with minimal motivational features. The fully gamified condition had one of the shallowest improvement slopes and none of the conditions made any difference on subjective motivation scores. However, even the minimally gamelike version still featured simplistic graphics and displayed a player score at the end of the block. It is possible that even this minimal gamification was enough to induce increased motivation and that adding “distracting” game elements such as persistent score display may have a negative impact on performance by inducing unneeded stress or new cognitive demands [34].

One of the most theoretically driven games in our review was *Watermons* [38], which included many motivational features aimed at delivering a sense of player autonomy and competence. The task-switching training was embedded within a rich storyline and graphically enhanced theme. They found that these gamelike features increased the effect of training, reducing reaction times and switch costs, compared against a non–gamelike version of the training. Participants were also more willing to perform training when in the gamelike condition.

Miranda and Palmer [36] used a visual search task with 2 forms of reward for fast and accurate responses: sound effects and points. They found that sound effects led to increased reaction times, potentially due to attentional capture and did not improve scores of subjective engagement. Points appeared to have no effect on data and boosted subjective engagement scores. These results highlight the delicate nature of designing gamified cognitive tests because something as innocuous as a few sound effects had deleterious effects on participant performance.

Finally, Hawkins et al, [9] compared gamelike versions of 2 decision-making tasks against nongame counterparts. No difference between the data collected by the gamified versions and the nongame versions was found. Subjective ratings indicated that both versions of both tasks were equally boring and repetitive, but that the gamelike versions were more interesting and enjoyable. Given the relatively large combined sample size of these studies (N=200), they provide good evidence that game mechanics can be included in cognitive tests without invalidating the data and with the desired effect of increasing motivation.

## Discussion

### Principal Results

We identified 7 reasons why researchers use gamification in cognitive research. These include not only the “traditional” applications of gamification such as increasing long- and short-term engagement with a task but also more clinically related reasons such as making tasks more interactive to enhance the effect of cognitive training. Several studies aimed to reduce test anxiety and optimize performance in groups that traditionally dislike being tested, particularly electronically, such as elderly people and children. By hiding the test behind a novel interface and gameplay, the target audience might feel more comfortable.

We saw several games aimed at training and testing people with ADHD, and overall, these games appear highly engaging to users, in some cases, even increasing the time spent training. Gamified tasks may be valuable for assessing ADHD patients as computer games are particularly appealing to them: with rapid rewards, immediate feedback, and time-pressure being exactly the type of stimulus the ADHD brain craves [64,65]. The dopaminergic system is thought to be abnormal in ADHD [66,67]. However, it is thought that playing video games can facilitate the release of extrastriatal dopamine, which plays a role in focusing attention and heightening arousal [68,69]; this may improve player performance and motivation. Nevertheless, as Delisle and Braun discuss [23], we must be cautious that liberal use of game mechanics does not reverse the very deficit we are hoping to measure.

One of the primary reasons that psychologists are keen to utilize gamification is to increase performance and motivation in research populations. The results of references [9,17,18,38], show that gamified tasks can be used to improve motivation, while still maintaining a scientifically valid task. However, Katz et al [34] and Miranda and Palmer [36] highlight that this can be difficult balancing act, with several game mechanics having unforeseen deleterious effects on performance. If gamified psychological tasks are to become common in the future, further research is required to disentangle the impact of specific game mechanics on task performance, as these studies have already begun to do.

### Differences Between Training and Assessment Tasks

Training games typically contained many features and were similar in appearance to commercial video games. Cognitive training normally requires several sessions to be effective, and as a result, training tasks need to be engaging enough to play for many hours. 3D graphics were quite prevalent, as was the use of avatars, points, levels, and dynamically growing game worlds (see Supplementary Figure 1 in Multimedia Appendix 2). Long-term goals which had to be completed over repeated sessions were also common and served to sustain engagement over a long period. In contrast, assessment games were simpler, predominantly using 2D graphics, sound effects, score, and theme to create the appearance of a game. Several games simply presented themselves as “puzzles” which the participant had to complete. Tasks of this nature represent gamification at its simplest, but they were well received by users, implying that minimal gamification is better than no gamification. The simplicity of gamification employed is likely due to the constant tension between creating an engaging task and the risk of undermining the task’s scientific validity: including unknown game mechanics might have deleterious effects on the data collected.

### Validating Gamified Tasks

We found heterogeneous standards for validating gamified tasks. Typically, cognitive assessment games were validated rigorously, using correlation with similar cognitive tasks and factor analysis to determine whether they were performing as expected. Many training games used a gamified task only, meaning the effect of gamification cannot be dissociated from the effect of the intervention. Sample sizes were small in nearly all of the studies we reviewed, and there was little consideration of statistical power when sample sizes were decided upon, with only 5 of 33 papers describing a power calculation. Gamified cognitive tests are novel scientific instruments and must be validated as such. Small pilot studies, followed by larger validation studies including assessment of test–retest reliability, and internal and external validity of the measures taken by the game are needed [70]. Regarding cognitive training, ideally gamified training should be treated as an intervention and so the current gold standard of a blinded randomized control trial is appropriate [71]. In both testing and training, we would recommend the use of at least 2 controls: a standard cognitive task designed to produce the same output measures/training effect as the newly gamified task and a nongamified version of the gamified task, built on the same software platform and identical in function/interaction, with all game mechanics removed.

### Limitations

One limitation of this review is the necessity of a narrow scope. Gamification in psychology and psychiatry is a rapidly growing field, hence we decided to focus specifically on “cognitive training and testing.” This has resulted in some papers being excluded on the subjective basis of not being “cognitive research.” Nevertheless, to counteract this subjectivity, papers were only included in the review on consensus from both reviewers (JL and EE), and a 20% selection check was performed by EE on papers from the title-screening stage onward. An additional consideration is that many of the studies reviewed were of a preliminary nature, and as such, the findings reported here should be considered tentative.

### Conclusions

As discussed by Hawkins et al [9], it has often been suggested that gamified cognitive tasks may result in higher quality data and more effective training, simply by virtue of heightened engagement. Our review found no evidence to support that gamified tests can be used to improve data quality, either by reducing between-subject noise or by improving participant performance, and there were some indications that it may actually worsen it [17,18,34,36]. We did, however, find some evidence that gamification may be effective at enhancing cognitive training, but we must take these positive training findings tentatively due to numerous methodologic problems in the studies that we reviewed.

Irrespective of whether gamification can improve data or enhance training, there are still many reasons why gamelike tasks may play an important role in the future of cognitive research. Gamified cognitive tasks are more engaging than traditional tasks, thus making the participant experience less effortful and potentially reducing drop-outs in longitudinal studies. Furthermore, despite concerns that some commonly used game mechanics might reduce participant motivation [72] (such as by having a visibly low score [73]), we saw no evidence that this was the case. Indeed, gamification was reported as both motivational and positive by study authors and participants alike. Gamelike tasks may also reduce feelings of test anxiety and allow alternative interfaces to cognitive tests that would otherwise be difficult to deliver in certain populations. The results of this review also show that it is possible to design gamified cognitive assessments that validate well against more traditional measures, providing that caution is taken to avoid developing mixed-domain measures or masking deficits of interest.

As cognitive research begins to move out of the laboratory and onto personal computers and mobile devices, engagement will be the key to collecting high-quality data. Gamification is likely to play an important role in enabling this change; but further research and more rigorous validation is needed to understand the delicate interplay between game mechanics and cognitive processes.

## Appendix

Multimedia Appendix 1 Five tables detailing coding and categorization in the main manuscript. Table 1 lists the reasons for using gamification in cognitive training and testing. Table 2 categorizes games by the age group they were aimed at. Table 3 lists games by training or testing category. Tables 4 and 5 list games by the cognitive domains which they targeted.

Multimedia Appendix 2 Additional information on the papers covered by this review that is highly detailed and intended for further analysis in future papers or for other researchers to assess themselves. It contains short text-based descriptions of all 31 games and a screenshot where the paper included one. There is also a detailed breakdown of the game mechanics in each task.

## Abbreviations

ADHD: attention deficit/hyperactivity disorder
EF: executive function
