# Supplementary material for: Gamification of Cognitive Assessment and Cognitive Training: A Systematic Review of Applications and Efficacy
Source: JMIR Serious Games. 2016 Jul 15;4(2):e11. doi: 10.2196/games.5888 (PMC4967181; doi:10.2196/games.5888)
Supplement: Multimedia Appendix 1 [file games_v4i2e11_app1.pdf]

Gamification of cognitive assessment and cognitive training:
A systematic review of applications and efficacy
Supplementary Information 1

Supplementary Table 1: Reasons for using gamification in cognitive training and testing

**Reason**	**Games**	**Count**
To increase participant motivation	Space Matrix, Card-pairing, Wii Tests, Retirement Party, Supermecha, BAM-COG, ABMT App, MACBETH, TENI, The Great Brain Experiment, Megabot, Groundskeeper, Playmancer, Tap the Hedgehog, Braingame Brian, GAME	16
To increase usability/intuitiveness for the target age group	Groundskeeper, Tap the Hedgehog, TENI, Whack-a-mole, Smart Harmony, Space Matrix, WMTrainer, Kitchen and Cooking, Eldergames, Wii Tests, BAM-COG	11
To increase long-term engagement	BAM-COG, ABMT App, The Great Brain Experiment, WMTrainer, Eldergames, Card-pairing, Playmancer, Braingame Brian	8
To investigate the effects of game-like tasks	EM-Ants, Ghost Trap, Visual Search, Watermons, Megabot, WMTrainer, GAME	7
To stimulate the brain	Eldergames, Wii Tests, Whack-a-mole, ABMT App, MACBETH, Kitchen and Cooking	6
To increase ecological-validity	Playmancer, MACBETH, Kitchen and Cooking, VAP-M, Groundskeeper, Tap the Hedgehog	6
No clear reason given	Space Code, Xcog, Cogoland, Neuroracer, Shapebuilder	5
To increase suitability for the target disorder	Megabot, Retirement Party, Supermecha, Braingame Brian	4

Supplementary Table 2: Games categorised by the age group they were aimed at

**Age Group Targeted**	**Game**	**Count**
All ages	EM-ANTS, Ghost-Trap, MACBETH, Playmancer, Shapebuilder, Space Code, Space Matrix, The Great Brain Experiment, Visual Search, Xcog, GAME	11
Children	Braingame Brian, WMTrainer, Groundskeeper, Megabot, Supermecha, Tap, TENI, Watermons, Cogoland	9
Adults	ABMTApp, Retirement Party	2
The Elderly	BAM-COG, Eldergames, Neuroracer, Wii Tests, Kitchen and Cooking, VAP-M Whack-a-mole, Smart Harmony, Card-Pairing	9

Supplementary Table 3: Games listed by category: testing or training

**Category**	**Game**	**Count**
Training	Supermecha, Braingame Brian, Cogoland, Xcog, Smart Harmony, ABMTApp, WMTrainer, MACBETH, Playmancer, Neuroracer, Card-Pairing, Watermons, GAME	13
Testing	Space Code, Space Matrix, Eldergames, Wii Tests, Retirement Party, Groundskeeper, EM-Ants, Tap the Hedgehog, BAM-COG, VAP-M, The Great Brain Experiment, Megabot, Whack-a-mole, Visual Search,	17

	Shapebuilder, TENI, Ghost Trap	
Both	Kitchen and Cooking	1

Supplementary Table 4: Testing games categorised by target cognitive domain

**Domain**	**Game**	**Count**
Working Memory	Space Matrix, Eldergames, Wii Tests, Retirement Party, Tap, BAM-COG, VAP-M, The Great Brain Experiment, Whack-a-mole, Shapebuilder, TENI, Kitchen and Cooking, Megabot	13
Decision Making	Eldergames, EM-Ants, VAP-M, TENI, Ghost Trap	5
Executive Functions	Retirement Party, Tap, BAM-COG, VAP-M, The Great Brain Experiment, Whack-a-mole, TENI, Kitchen and Cooking, Megabot	9
Processing Speed	Space Code, Space Matrix	2
Attention	Eldergames, Wii Tests, Groundskeeper, The Great Brain Experiment, Visual Search, TENI, Kitchen and Cooking	7
Inhibition	Retirement Party, Groundskeeper, The Great Brain Experiment, Whack-a-mole, TENI, Kitchen and Cooking, Megabot	7

Supplementary Table 5: Training games categorised by target cognitive domain

**Domain**	**Game**	**Count**
Working Memory	Supermecha, , Braingame Brian, Xcog, Smart Harmony, WMTrainer, Playmancer, Watermons, Card-Pairing, GAME	9
Decision Making	Playmancer	1
Executive Functions	Braingame Brian, Xcog, Smart Harmony, MACBETH, Neuroracer, Kitchen and Cooking, Watermons	7
Attention	Smart Harmony, ABMTApp, Kitchen and Cooking, Card-Pairing	4
Inhibition	Braingame Brian, Smart Harmony, MACBETH, Playmancer, Kitchen and Cooking, Watermons	6
