# Supplementary material for: Gamification of Cognitive Assessment and Cognitive Training: A Systematic Review of Applications and Efficacy
Source: JMIR Serious Games. 2016 Jul 15;4(2):e11. doi: 10.2196/games.5888 (PMC4967181; doi:10.2196/games.5888)

Gamification of cognitive assessment and cognitive training: A systematic review of applications and efficacy

Supplementary Information 2

Supplementary Table 1: Overview of games from included studies

Where minigames that warranted further detail, they are listed under the main game with the its name as a prefix

**Minigames**	**Description**	**Picture**
BAM-COG	Four puzzle games to measure WM, visuospatial short-term memory, episodic recognition memory and planning. Fairly simply 2D graphics with sounds. Each game has a practice trial followed by testing levels.	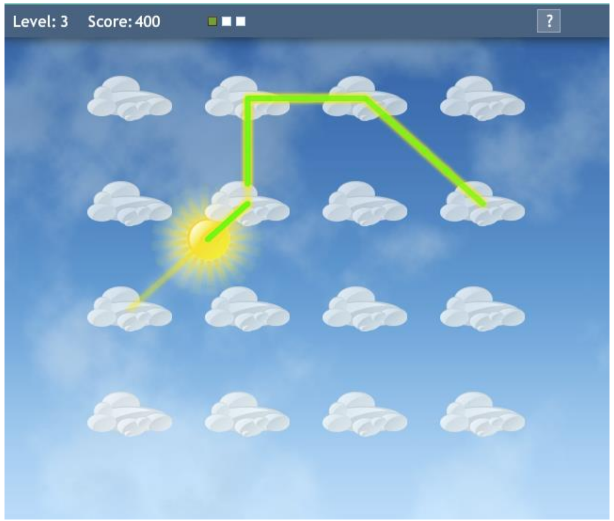
BAM-COG: Conveyor Belt	Focuses on WM: A grocery list is displayed on the screen. After 1 second products start scrolling along the top of the screen. Participants must select only those products that are on their list, and the correct amount of each.	
BAM-COG: Sunshine	Focuses on Visuospatial WM: Patterns appear in a 5x5 matrix then dissolve gradually. Once the pattern has disappeared participants are asked to reproduce it in the same order as it initially appeared.	
BAM-COG: Viewpoint	Focuses on Episodic Memory: A 5x5 matrix filled with stimuli is presented. The participant gets three seconds to memorize the pattern before it disappears from the screen. After three seconds three possible answers appear from which the participant is to pick the pattern that was previously shown.	
BAM-COG: Papyrinth	Focuses on Planning: A scrambled route is presented in a grid to the participant. The participant has to complete the route so their character can move from the start to the finish. Clearing the route is done by sliding the columns and rows in such an order that all pieces of road end up connected to one another.	
Braingame Brian	An EF Training game. Each training session takes 40-50 minutes, with 30 min of training minigames and 10-20 min of world exploring. Contains an extensive 3D world with multiple areas and characters. All the characters have problems which the player character, Brian, helps to solve by playing minigames and inventing clever machines. After each minigame the difficulty level of the game is adjusted to match the player’s level of performance. Braingame Brian is designed to be played once through with a therapist as a guide, before being given to the participant to take home.	
Braingame Brian: WM	Focuses on Visuospatial WM: A 4x4 matrix is presented on the right hand side of the screen. A random pattern appears on the screen which the player must reproduce. For	

	every correct reproduction the player is rewarded by revealing more of the blueprint for the reward which will be constructed when the task is complete.	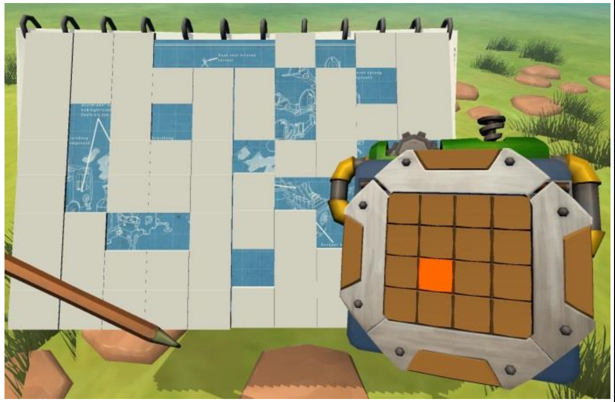
Braingame Brian: Stop Task	Focuses on Inhibition: A large machine with two lights and a gauge is displayed on the screen. When a light is lit blue and the gauge is in the appropriate range then the player must rapidly respond by pressing the appropriate key (go trial). However, when a light is lit red, the player must inhibit their response (stop trial).	
Braingame Brian: Switch Task	Focuses on Cognitive Flexibility: Another large machine with a conveyor belt is displayed on the screen; construction parts appear on the conveyor belt and must be sorted by the player as quickly as possible. The attribute (shape/colour) to sort on is displayed at the top of the machine, and periodically changes.	
Card-Pairing	In this game the participant must concentrate their attention in order to open and close the cards on the screen. Their ‘brainscore’ is recorded on the screen, as is their current level of completion.	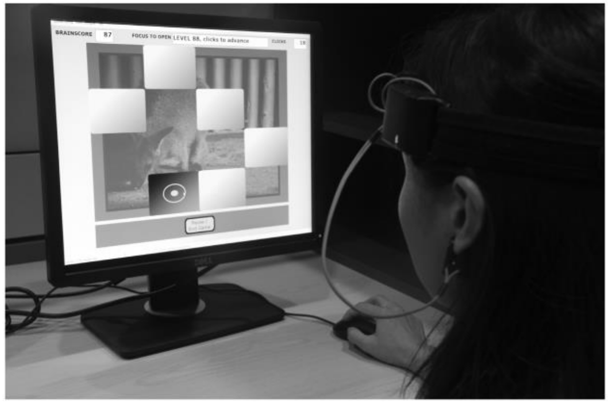
Cogoland	Biofeedback training game using an EEG headband. Hardware is calibrated to detect the player’s most attentive state using a pre-test stroop task. 3D world which the player can explore using their avatar, whose movement speed is controlled by the intensity of the players’ concentration. Players would complete three minigames involving speed, control and memory, which took 30 minutes including breaks.	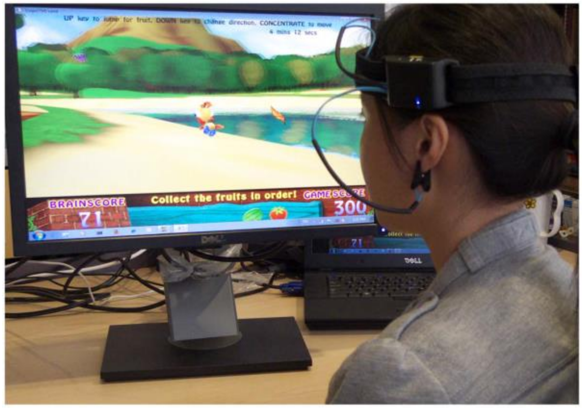
Cogoland: Race	Focuses on Concentration: The goal of the first level is to make the avatar run around an island in the shortest time possible. The avatar follows a predetermined route, so all the player has to do is concentrate.	
Cogoland: Jump	Focuses on Concentration and Dual Tasking: The goal of the second level is to collect a series of fruits floating in the air while the avatar navigates through a colourful town on a pre-determined route. When the avatar is underneath a fruit the player has to press a specific key on the keyboard to make the avatar jump to collect the fruit. The	

	aim is to collect as many fruits as possible within a given timeframe, after which the number of fruits collected is entered into a personal logbook.	
Cogoland: Order	Focuses on Concentration and Dual Tasking and WM: The third level is similar to the second level except that the fruits must be collected in a specific order.	
Eldergames	Mixed-reality tabletop game with a top-down projected table and large pens as input devices. Designed to facilitate training of cognitive faculties in the elderly. Allows a maximum of four players to play together, either collocated or via the internet. The game is divided into two sections: the Memo-game, which serves as a bridge between the minigames, and the Minigames, which are designed to train specific cognitive abilities.	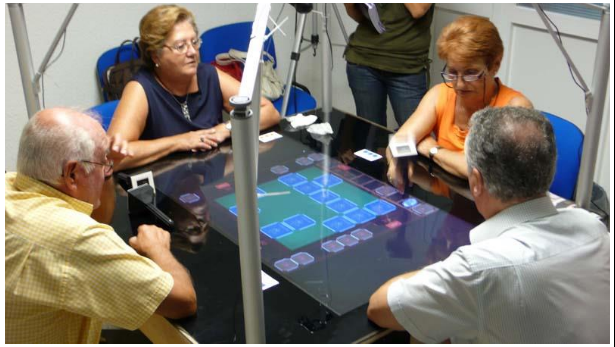
EM-Ants	Tests visual discrimination and decision making. EM-Ants or ElectroMagnetic Ants from a parallel universe are eating electromagnetic radiation all across the Universe. You have a spaceship and must halt their advance. The screen displays a grid of ‘galaxies’ which gradually populate with dots. One cell populates faster than the others. Detect which galaxy contains the ant queen by determining where they are breeding fastest and then shoot it with your laser. Very simple 2D game with minimal animation, but a strong theme.	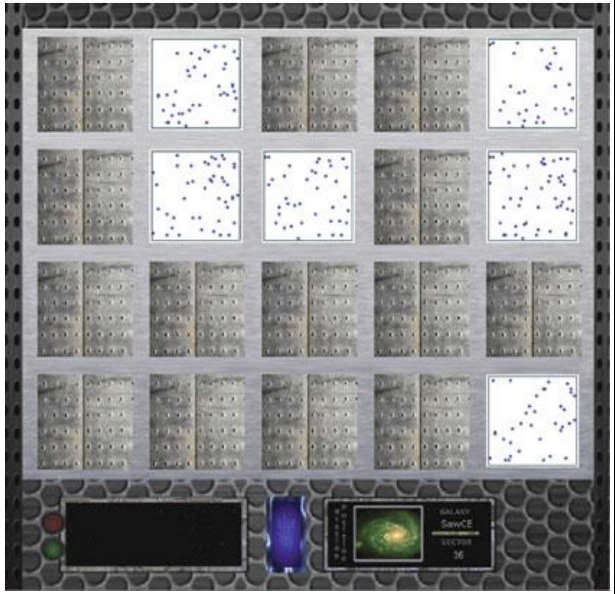

Ghost Trap	Tests decision making and probability estimation. Two corridors are displayed on the screen. Ghosts head down the corridor to the end where they enter the ghost trap; players must trigger the trap to catch a ghost. The distribution of ghosts in each corridor is different, so one corridor will catch more ghosts than the other. At some point the distribution changes and players must adapt to continue doing well. Half way through the game the lights go out and the corridors go dark: at this point the only way to estimate the distribution of ghosts is by catching them. Again the player must adapt to any changes in distribution as quickly as possible.	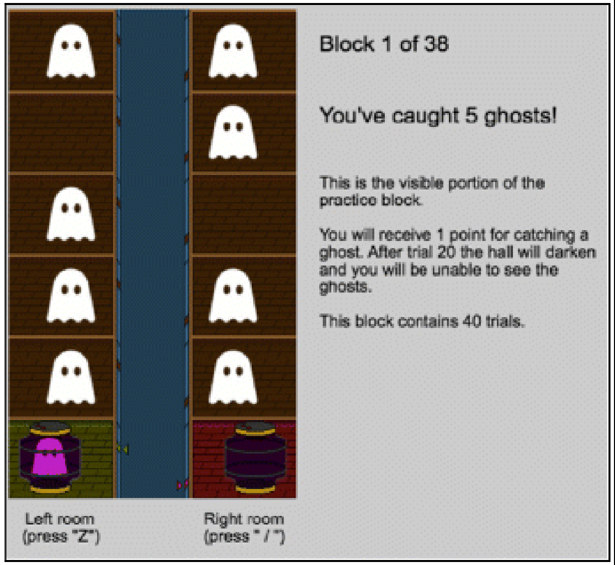
WMTrainer	Focussed on training WM. Essentially a spatial n-back task which presented participants with stimuli at one of six locations on the screen, at a rate of 3 s each. The player has to press a key each time the stimulus presented matched the location of the one presented n items previously, where n varies with difficulty. After 4+ errors the player loses a life, after losing three lives the player's n goes down by 1 next round.<br>Seven versions of the training game were developed in order to examine the effect of five different game mechanics: points, graphical theming, explanation of lives and levels, prizes, and end-of-session certificates. Overall the game was a fairly simple 2D game with minimal animation but a good quality graphical finish.	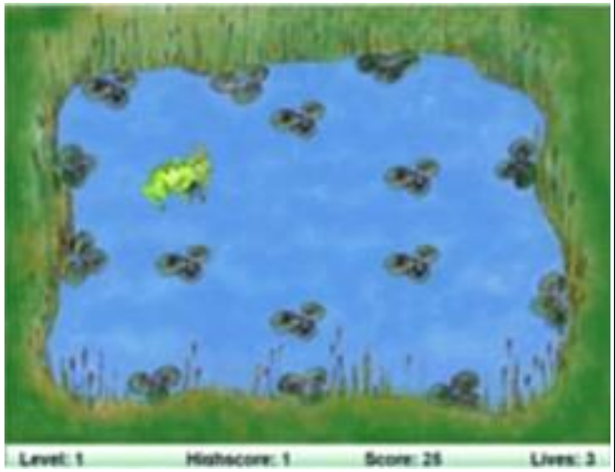

ABMTApp	Designed to modify attentional bias away from angry faces. The game is set in a grass field, and as a trial starts two animated characters appear at the top of the screen; one with a mildly positive face and one with an angry face. Shortly after appearing they burrow into a hole. The positively-faced character causes a path of grass to rustle behind it. The player must use their finger to trace the path of the rustling grass as quickly and as accurately as possible. Points were awarded on the basis of speed and accuracy. The game provided participants with feedback after each trial by presenting one of three possible “jewels” that varied in colour and accompanying sound.	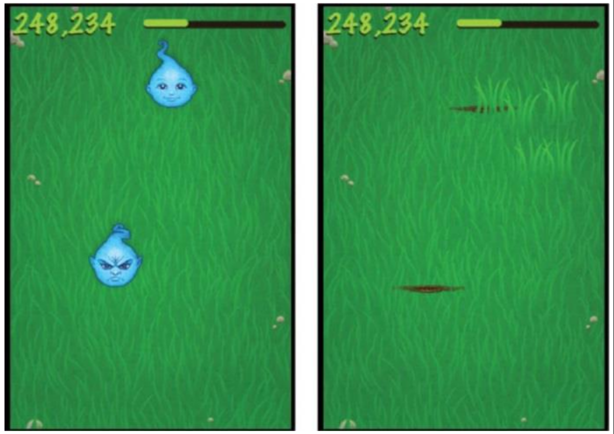
Groundskeeper	Machine-learning based ADHD diagnosis game. Uses physical ‘smart cubes’ with screens on one side and many internal sensors which can detect motion. The cubes communicate wirelessly and can be synced to a computer that runs the game and collects data. Groundskeeper is played using four cubes and a placement board; it is essentially a whack-a-mole game. One cube is always used as a mallet to hit the game’s targets: gophers that appear on the screens of the other cubes. Occasionally other stimuli will appear on the cubes, the aim of the game is to ignore the distractors and hit the gopher as quickly as possible to produce a satisfying “bonk” sound.	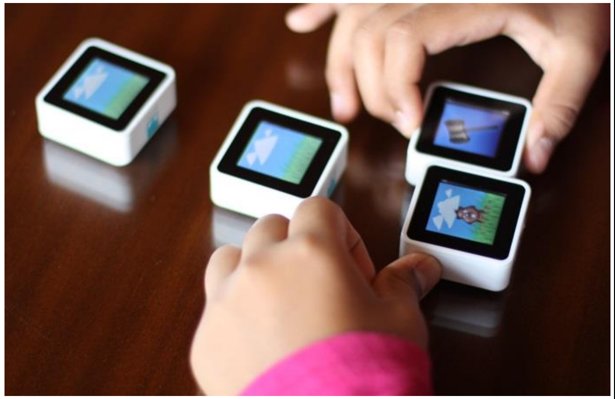
GAME	GAME is a gamified working memory training task, and is presented to the player as a personal quest of freeing one’s own brain areas (This is visualized by a coloured brain region which is visually overlaid by a lock). In order to achieve this goal the player must remember a multi-digit number while categorising “distractor equations” as true or false. Once they have categorised all the distractor equations they are asked to enter the original number in order to proceed to the next level. Once the player types in the correct numbers the lock opens and the formerly locked brain area is freed. The player receives visual feedback of correct (growing neuron animation) or wrong (shrinking neuron animation) answers depending on the result of the Test-phase.	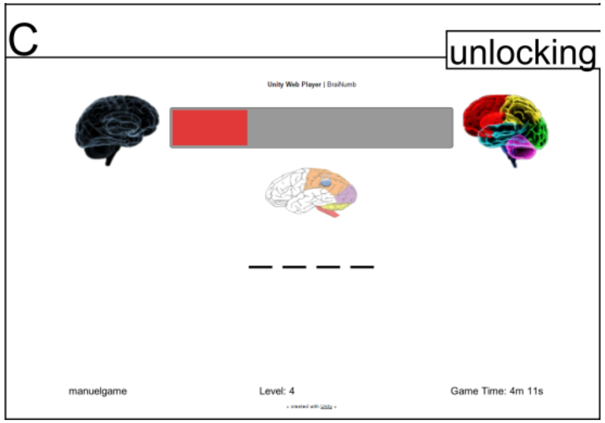

<table>
<tr>
<td>Kitchen & Cooking</td>
<td>Kitchen and Cooking is an Ipad game based on the idea of cooking four different recipes. Several minigames must be completed within the allotted time in order to complete each dish: selecting the correct ingredients from the fridge, planning cooking-actions and performing physical actions on the screen in order to do the cooking itself. Kitchen & Cooking uses a blend of 2D and 3D graphics and an interactive chef character as a helper. Each of the recipes is harder to cook than the last.</td>
<td>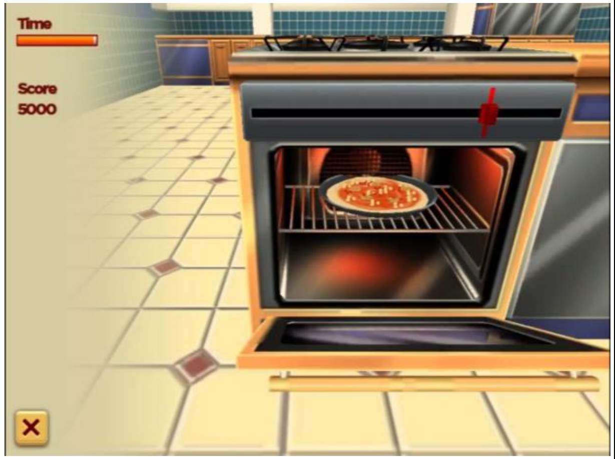
</td>
</tr>
<tr>
<td>MACBETH</td>
<td>Macbeth is a strategy/mystery game designed to modify cognitive biases such as fundamental attribution error and confirmation bias. Training is supplied within a game about stopping an impending terrorist attack by figuring out who the suspect is, the method of attack and where it will occur. Analysts gather information and work with AI characters to prove and disprove hypotheses. Through the game the player is presented with info on cognitive biases. Feedback is used to mitigate the impact of various cognitive biases on their decision about who will commit the crime.
The game itself is a 2D interface-based point and click game. The player can order investigations into certain locations and people, and they are presented with the evidence to analyse.</td>
<td>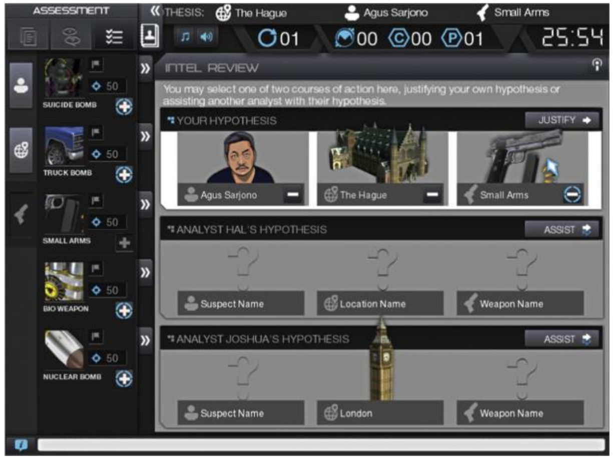
</td>
</tr>
</table>

Megabot	Megabot is a WM assessment game designed for children with ADHD. The basic WM training is the Corsi Block Tapping Task (repeating a demonstrated pattern on a 4x4 grid) but game elements are added including animation, gameplay, a storyline, upgrades and competition. The player must save the world from destruction by using their Megabot to drive out the evil robotic invaders. Levels are beaten destroying all occupying enemy-robots without taking too much damage.<br><br>In order to destroy an enemy-robot, complete an objective, or protect the player's Megabot from being damaged the player has to correctly recreate a WM-task sequence. The difficulty level of the game is adaptive with the sequences increasing or decreasing in length to match the player's skill. After 60 trials the evil-robots surrender and the player automatically wins.	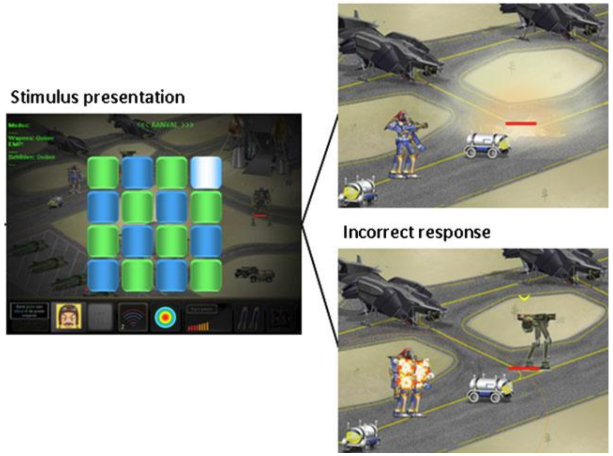
Neuroracer	Neuroracer is a 3D video game to challenge perceptual discrimination while the player is performing intense visuomotor tracking. It is a cartoony, 3D racing game with a random track featuring left and rights turns, as well as hills. While the player is driving their car they must also respond (and occasionally inhibit) rapidly to signs that appear at the top of the screen by pressing the correct key. The car was able to freely on the screen, and hence the player had to continually maintain control in order to keep their vehicle on the road.	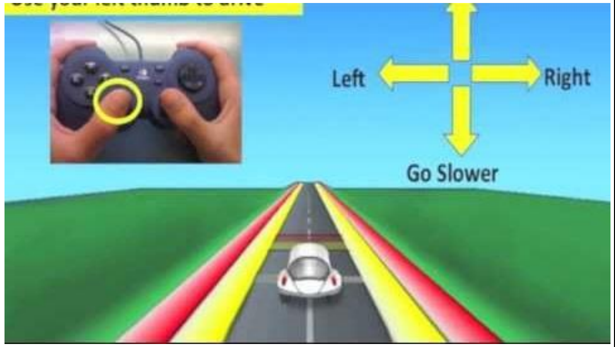
Wii Tests	Designed for testing elderly people cognitive function. Addresses attention, memory and motor control. Built on the Nintendo Wii games console and controlled with a Wii mote. All games are simplistic 2D games with no story or points.	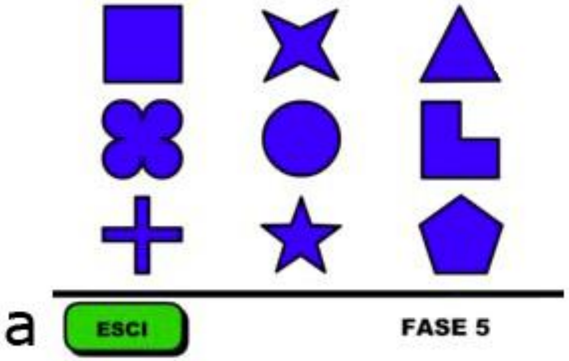
Playmancer	Playmancer is a fully realised 3D biofeedback training game. During the video game, the player is confronted with several challenging situations in the form of 3	

<table>
<tr><td></td><td>minigames. As the player completes the various tasks of the game they can advance to a higher level of difficulty. In-between the minigames the player may explore a virtual island world in a custom designed avatar.</td><td rowspan="4">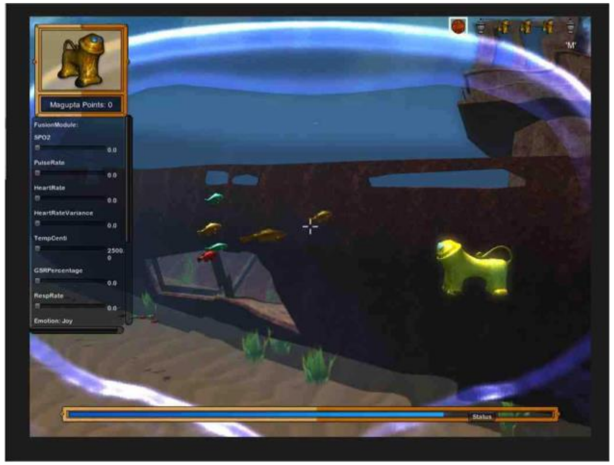
</td></tr>
<tr><td>Playmancer: Treasures of the Sea</td><td>This game features an underwater environment in which the player swims under water, gathering artefacts and balloon fish. The player has to maintain their oxygen level in order to keep on playing. This task is made more difficult if there is high arousal as an increase in heart rate, pulse rate, respiration rate, and facial expressions of anger emotion produce more difficulties (e.g. it is harder to catch fish and the oxygen runs out faster).</td></tr>
<tr><td>Playmancer: Face of Chronos</td><td>In this game the player must control an avatar as they climb up a cliff. High arousal increases the number of obstacles and so, in order to do well, the player must keep their heart rate low while navigating the cliff-face.</td></tr>
<tr><td>Playmancer: Sign of the Magupta</td><td>This is a relaxation game in which constellations of stars are drawn corresponding to the level of calmness of the player (based on breathing parameters). Slow breathing leads to a pathway connecting stars with each other, with different levels of calmness revealing different constellations.</td></tr>
<tr><td>Retirement Party</td><td>Retirement Party is a complex, strategy-orientate puzzle game was can be used for testing and training ADHD patients. However, it was designed for the express purpose of testing various models of ADHD. It takes the form of an executive function test realistically simulating an occupational environment, with an incidental memory task at the end of the procedure. The aim of the game is to play four days in the life of a diabetic home-based working accountant who has to self-inject insulin at four precise times of the day, has to answer the phone and go to bed at midnight, has to do accounting-like activities, and has to organize a retirement party for a colleague. The player must complete the simulation as quickly as possible.

The main display frame of the simulation consists of a grid of 30 actions, randomly displayed, all relevant to organizing the retirement party, which have to be selected by the participant in a logical order. When this task is completed, an unannounced memory task is imposed. The game requires the player to quickly execute over a thousand constrained key presses, answer multiple simultaneous high-order executive demands, all the while remaining constantly aware of ongoing virtual time so as to complete each assignment at the right time or at the right moment.</td><td>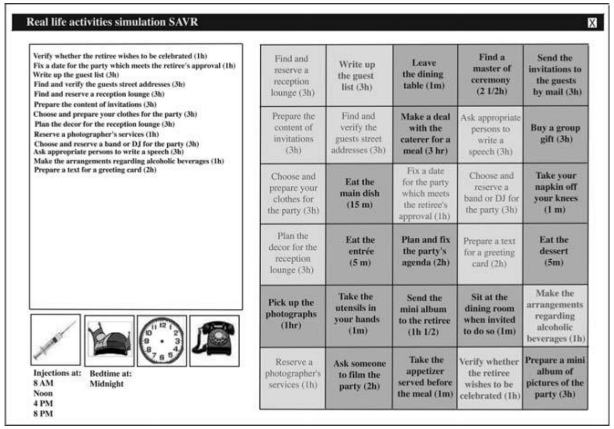
</td></tr>
</table>

Shapebuilder	Shapebuilder is a web-administered cognitive ability test in which players are asked to remember the order and spatial position of a series of coloured shaped. The task is automatically scored and takes under six minutes to administer. Players view a 4x4 grid squares: a sequence of 2-4 coloured shapes appears and the player must remember the location of each item, the shape of each item, the colour of each item, and the order that the items appeared in.<br><br>The player then recreates the sequence by dragging shapes to the grid from the ‘Shapebuilder’ toolbar. The game has varying difficulty levels and players receive immediate feedback about the accuracy of each item: correctly placed shapes are awarded points. The more correctly placed items in a row, the more bonus points are awarded. This exponential scoring rule provides motivation for participants try to remember the entire sequence of shapes, rather than focusing on one or a few shapes.	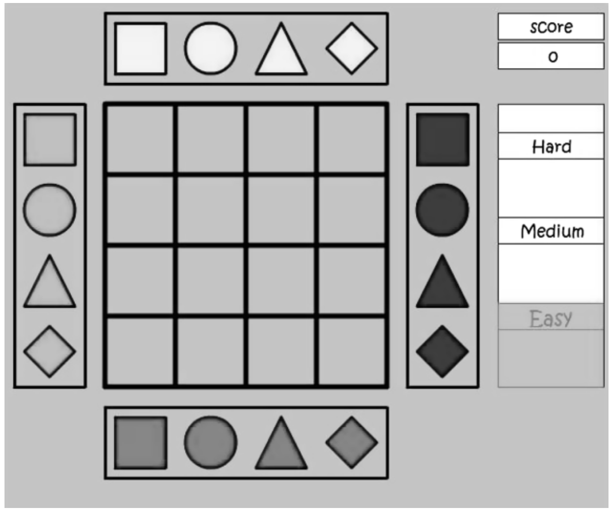
Space Code	Space Code is a Symbol Digit based test. The main screen shows a spaceship cockpit with a numeral response grid. The player must match the spaceship on the viewscreen with a spaceship on the selection bar and press the corresponding number as far as they can. When they do so, a laser is launched and the enemy ship is destroyed in an explosion, if they match incorrectly, their own ship takes damage and the response pad is disabled for a few seconds.<br><br>Bonus stars provide extra points on some levels. The game is 2D but includes sound effects, background music and animation, and well as a point scoring system.	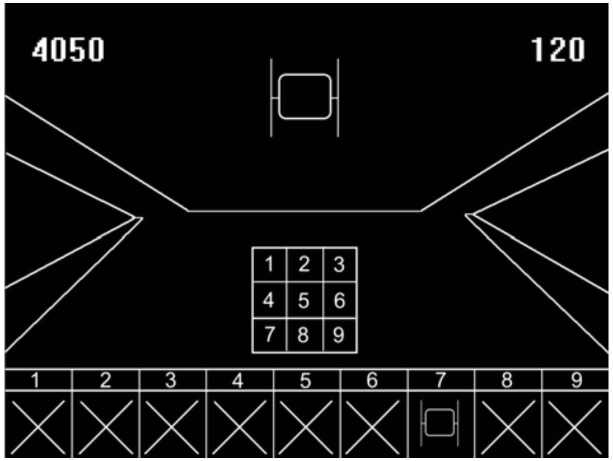
Space Matrix	Space Matrix is an updated version of Space-Code that also tests WM, so the two games are very similar. In Space Matrix the player is shown a cockpit with approaching enemy spaceships, but their control panel contains a 5x5 grid with a dot in additional to the usual digit-symbol taskbar. The game plays like Space Code except that the dot in the grid represents the sector of space that the player is operating in. The sector changes every 7 secs and every 2-4 sectors the player must report which sectors they have been in. Score bonuses given for this part of the test only. The background music was removed and replaced with humming.	

Smart Harmony	Smart Harmony is a team game that must be played in a group of seven. Each player is given a ‘smart stick’ that is synchronised to one of the seven musical notes. The team sits around a screen which shows the musical notes of the song currently being played. When the timing bar passes the corresponding note in the song, the relevant players must shake their sticks. Score is shown as individual scores and a combined score.	
Supermecha	Supermecha is a WM training task designed for improving the symptoms of kids with ADHD. The training is a simple visuospatial WM training task in which the player has to reproduce sequences of randomly lit-up squares in a 4x4 grid, however, the task is presented a computer game. The story is that the player has to save the world from an evil group of robots, named Mechas, which have taken control and invaded villages. The player must take control of a ‘‘good’’ robot, the Supermecha, fight the evil robots. The game consists of three levels and in each level several villages could be ‘‘re-conquered.’’ Once a village has been entered by the Supermecha, the evil robots can be shot: but the shot’s success is determined by the player’s performance in a WM task.<br><br>The WM training is described as ‘coordinates of the bullets’, which have to be remembered correctly in order to be able to attack the evils robots successfully. The Supermecha can also be attacked by the evil robots and successfully reproducing a WM pattern in this instance rewards the player with a bonus, with 5 bonuses providing an extra shot against the robots. In general, it takes 30min to play a whole training session. The game itself is a 2D and isometric with sound effects and animation.	

Tap the Hedgehog	Tap the Hedgehog is a fairly simple series of tests that utilise a tangible interface in order to perform cognitive testing and training. It is aimed at young children. The TagTiles interface is a small board which can detect the presence of pieces placed upon its surface, and can be overlaid with a 'playmat' in order to customise its interface. In this game the playmat is countryside themed, with little pictures of hedgehogs on every square. Despite all the tasks delivered on the TagTiles interface essentially being variations on the Corsi-block tapping task, the game manages to make sense of these tasks within a storytelling context. Furthermore the players are aided by two hedgehog characters that use voiceovers in order to guide the player through the game.<br><br>Patterns are displayed on the board using backlights and the player must interact with them using the 'pawn' counters at the top of the board. Sometimes the correctly coloured pawns must be matched to the correctly coloured squares as an additional task requirement. As every task is completed the player earns points which can be saved up in order to 'hedgehog houses', bonus points are awarded for speed and accuracy. This serves as a form of long-term goal for the player.	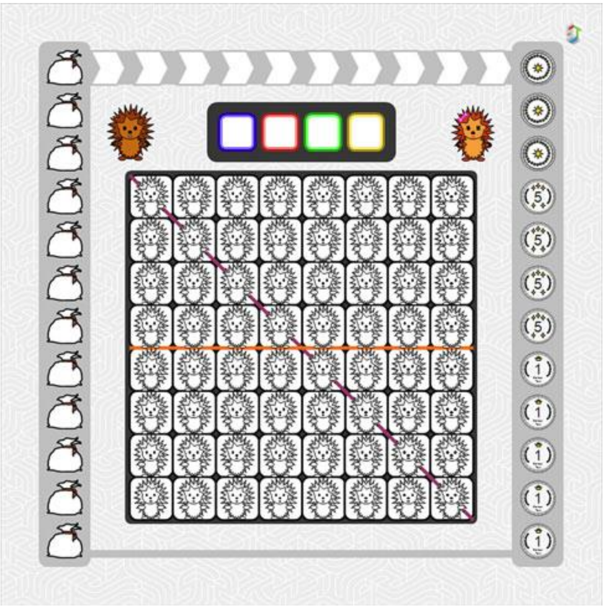
TENI	TENI is a cognitive profiling battery aimed at assessing children from 3-9years. It consists of 10 minigames which test different skills. Some of these tests require an experimenter to deliver or assess the task. A variety of game-types and graphics are used, but most tasks try to construct a reason for why the player must act in a certain way. Tasks include: spot the difference, sustained attention, rapid naming, block-tapping, etc. TENI is delivered on a touchscreen device.	
The Great Brain Experiment	The Great Brain Experiment is the first gamified cognitive test to be deployed on the Android and IPhone app stores. Its purpose is to gather normative cognitive data from users taking the tests in everyday environments. It originally consisted of four gamified cognitive tasks that used stylized 2D graphics and a soundtrack to draw the users in. Once a task is completed, players can compare their cognitive scores against those of other users.	
The Great Brain Experiment: WM Task	In the working memory (WM) game, players must remember the positions of red circles that appear in different positions on a 4x4 grid. Sometimes there are distractor circles that appear in yellow. Once the pattern has been displayed, the player must repeat the pattern back.	

The Great Brain Experiment: Attentional Blink Task	In the attentional blink task, participants are required to identify the second of two target images in a rapid serial visual presentation. The player is instructed to watch for a target category of objects, such as “birds”. The play-screen shows a pulldown projection screen, an old-fashioned projector in the foreground, and a cartoon scientist character indicating that participants should pay attention to the projection screen. A series of images, that do not match the target, are rapidly presented on the projector, with two target images amongst them. The presentation is followed by a forced choice task in which the player must identify the second target picture they saw. The task is presented as a game through the inclusion of whimsical stimuli.	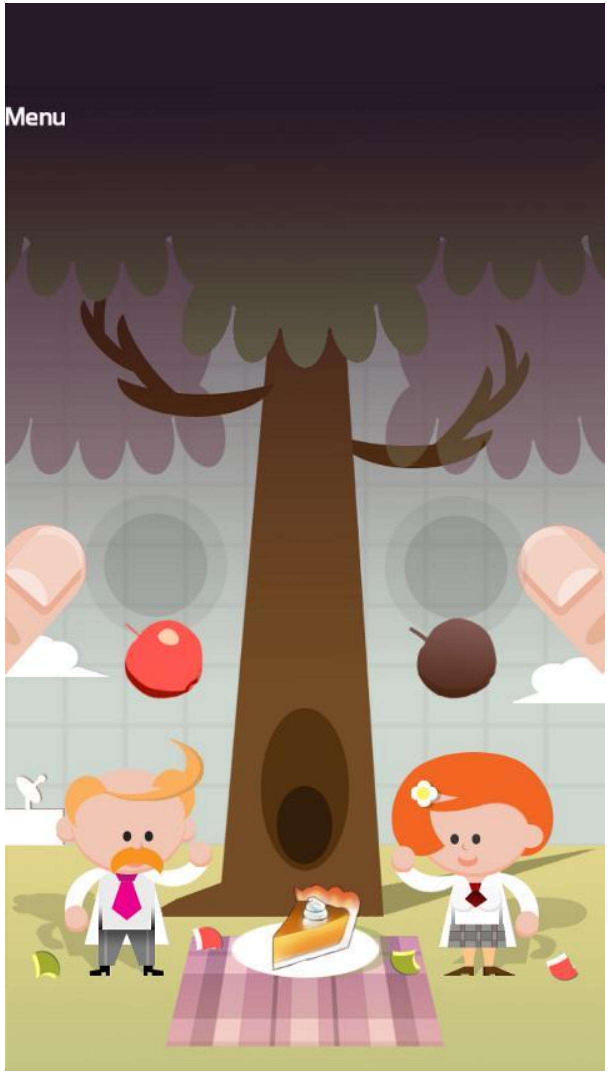
The Great Brain Experiment: Selective stop-signal Task	The second minigame is a selective stop-signal task designed to measure inhibitory ability. The game-screen shows two scientists having a picnic under a tree. When the test starts, two pieces of fruit appear at the top of the screen. After a delay, both fruits start to fall towards the bottom of the screen. The player must tap both sides of the screen as the fruit passed over a shaded area, however on some trials the fruit turns rotten as it falls, and so the player must only tap one side of the screen, inhibiting their response to the other side. On some trials a glowing circle appears around one of the fruits indicating to the player only that fruit could turn brown that trial.	
The Great Brain Experiment: Decision-Making Task	This decision-making minigame is designed to measure economic preferences including loss aversion and response to reward. The game is presented as a gambling task, where the player must choose between a gamble or a fixed prize. The player stars with a pot of points and they must aim to maximise their score. Participants are also asked every 2–3 trials to answer ‘How happy are you right now?’ by marking a point on a line.	

Visual Search	This visual search game is designed specifically to look at the effect of game mechanics on data from cognitive tests. As such, two versions of the game exist, one with points and one with sounds. The points-game involves gaining points on every trial, based on speed and accuracy of response. A high score was recorded throughout the duration of the study and upon achieving a new high score the player was rewarded by a fanfare and announcement. The current high score is constantly displayed in the corner of the screen. In the sound-game, arousing and pleasant sound effects are associated with point scoring, so the scoring of points becomes rewarding in itself, though no points are displayed. The points system utilises many features common to arcade games such as high score, player score, streak counter and bonus multipliers. Steaks are bonus points for every 5 correct trials in a row and increasing in value after that. Bonus multipliers occur at predetermined intervals. The game itself is very simple and features no graphical enhancement.	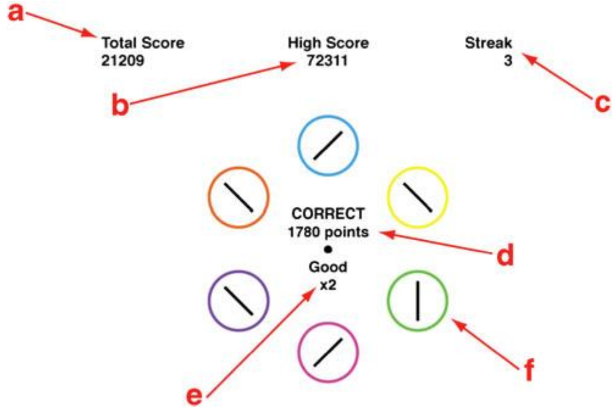
VAP-M	VAP-M is a realistic memory and planning test set within a virtual museum environment. Players are given pictures of five artefacts and written directions on how to locate them in the different halls of the museum. The player then has 2 minutes to try and memorize the pictures and directions before entering the game world with only 12 minutes to locate the artefacts. After a short break, the player then has a free recall test of any of the details of the artefacts.	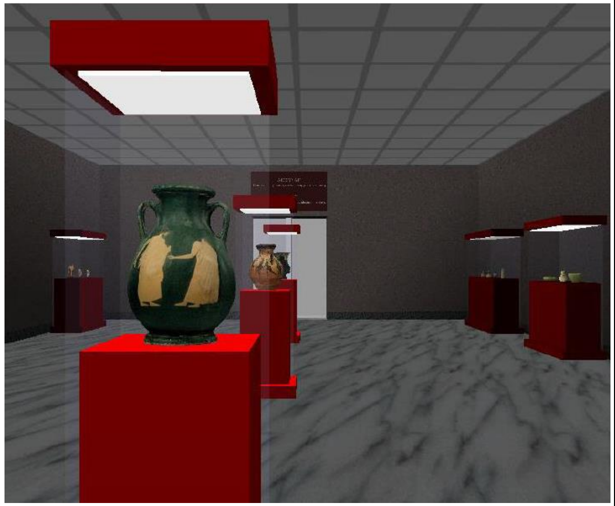

Watermons	In this game the player plays the role of an astronaut who has landed on a foreign planet. In order to repair their damaged spaceship they have to train the planet’s native Watermons to fight in Watermon battles to earn money. The main game itself is a task-switching test where the player must repeatedly decide whether the pictured Watermon was equipped with a net, energy shield, torpedo or energy ball. Which item they had to identify was dependent on the current task context, and the task context changed frequently. Fast and accurate responses rewarded experience points for that player’s Watermon, resulting in it growing stronger and more powerful. In between blocks players were shown how well they had performed, and whether they were a good enough trainer to achieve “tournament status”. If they entered a tournament and won they could choose a new Watermon to add to their team.	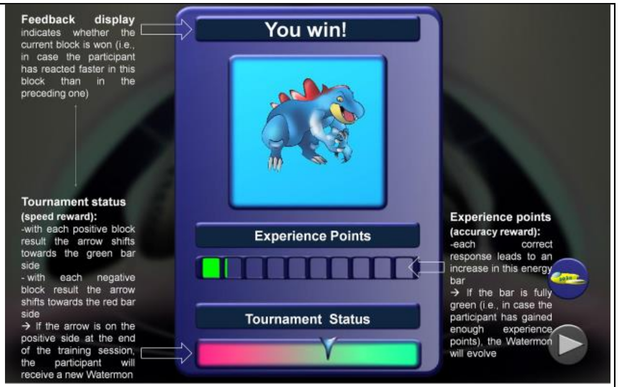
Whack-a-mole	This game was designed to be a flexible test with many adjustable settings allowing it to measure inhibition, executive functions and working memory. It is a simplistic 2D game to be played on a tablet. In the standard game, a mole appears from one of 16 holes in the ground, and the player must tap the mole as quickly as possible, while avoiding hitting the butterflies which occasionally appear.	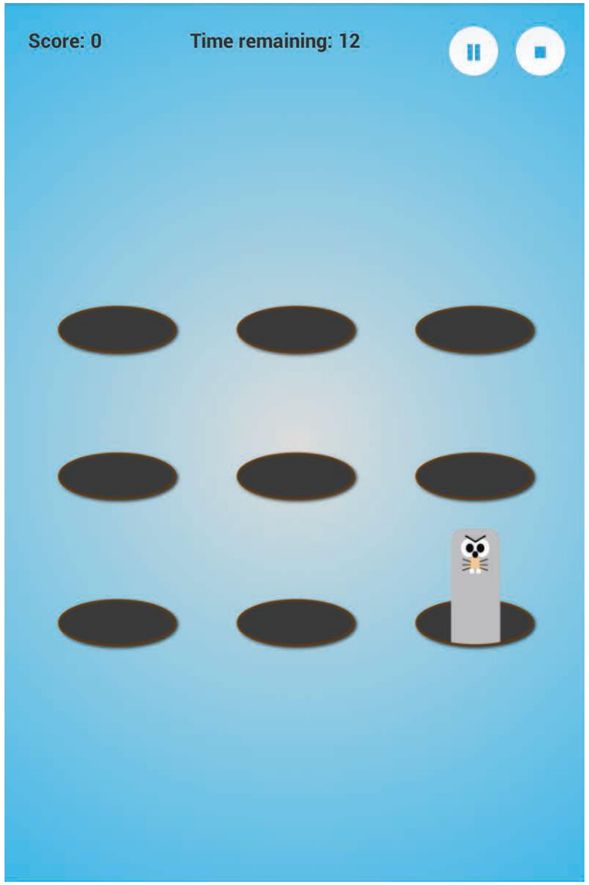

Xcog	Xcog is a battery of 16 tests designed to train visuomotor, memory, problem-solving and attentional abilities. Players have to control characters that face several adventurous challenges, such as rescuing a princess trapped inside a maze and protecting salads from hungry snails. The graphics are bright, cartoony and eyecatching.	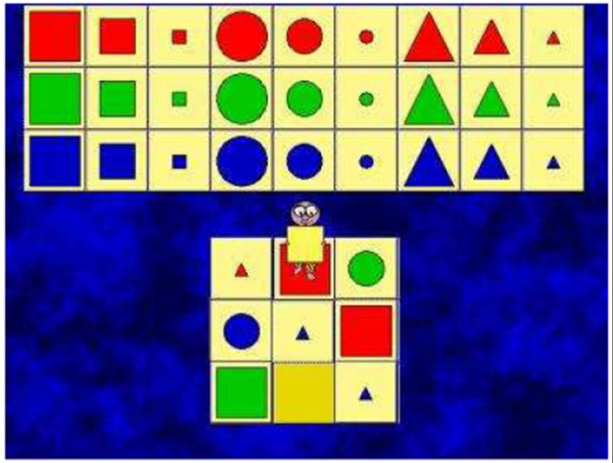

Supplementary Table 2: Categorised methods of validation for gamified testing tasks

**Validation Technique**	**Game**	**Count**
Compared against cognitive tasks/neuropsychological tests	Retirement Party, Tap the Hedgehog, Space Code, Space Matrix, Wii Tests, Groundskeeper, BAM-COG, Whack-a-mole, Shapebuilder, TENI, VAP-M, Kitchen & Cooking	12
Compared against a non-gamified task or similar control	EM-Ants, Ghost-Trap, The Great Brain Experiment, Visual Search, Megabot	5
Usability/Engagement assessed objectively	Space Code, BAM-COG, Whack-a-mole, Kitchen & Cooking	3
Engagement / Usability assessed subjectively	Tap the Hedgehog, Eldergames, EM-Ants, Ghost-Trap, Visual Search, Kitchen & Cooking	6

Supplementary Table 3: Categorised methods of validation for gamified training tasks

**Validation Technique**	**Game**	**Count**
Compared against a non-gamified task	Supermecha, WMTrainer, ABMTApp, Watermons, GAME	5
Pre and post training training-task performance assessed	Supermecha, Playmancer, Braingame Brian, WMTrainer, Neuroracer, Cogoland	6
Pre and post training behavioural questionnaires	Xcog, Braingame Brian, Playmancer, ABMTApp, Kitchen & Cooking, Cogoland	6

Pre and post training cognitive-task performance assessed	Neuroracer, Xcog, Supermecha, WMTrainer, MACBETH, ABMTApp, Playmancer, Kitchen & Cooking, Smart Harmony, Braingame Brian, Watermons, Card-Pairing	13
Usability/Engagement assessed objectively	Supermecha, WMTrainer, Kitchen & Cooking, Watermons	4
Engagement / Usability assessed subjectively	Supermecha, WMTrainer, Kitchen & Cooking, Card-Pairing, Watermons, GAME	6

Supplementary Table 4: Game mechanics used within cognitive training/testing tasks

Game mechanics are listed only if the paper clearly describes it or a figure clearly displays it

Game Mechanics	Game	Count
**Avatar**<br>The game gives the player a visible, humanoid character to control	Supermecha, Megabot, Xcog, Braingame Brian, Cogoland	5
**Background music**<br>The game includes background music	Playmancer, Megabot, Braingame Brian, Smart Harmony, The Great Brain Experiment, Space Code, Kitchen & Cooking	7
**Biofeedback**<br>Biofeedback is used either as a method of control or for player information	Playmancer, Cogoland, Card-Pairing	3
**Interactive Characters**<br>The game features characters (animals, humans, robots, etc.) which can be interacted with	MACBETH, Supermecha, Megabot, Braingame Brian, Kitchen & Cooking, Tap the Hedgehog	6
**Non-interactive Characters**<br>The game includes characters (animals, humans, robots, etc.) but they are mostly represented as static pictures and have little or no personality	WMTrainer, ABMTApp, Tap the Hedgehog, Groundskeeper, TENI, Whack-a-mole, Watermons	7
**Competition**<br>The game includes direct, live competition against other people	Smart Harmony, The Great Brain Experiment, Eldergames	3
**Complex motor control**<br>Playing the game demands precise motor control	Kitchen & Cooking, Wii Tests, Groundskeeper	3
**Dynamic world growth**	Supermecha, Megabot, Braingame Brian, Watermons	4

As the player plays the game, the virtual world changes permanently in response to their actions		
**Overarching Goal**<br>The player is given a goal which ties together all the minigames/levels and gives them a clear, long-term objective to achieve	MACBETH, Supermecha, Megabot, Braingame Brian, WMTrainer, Kitchen & Cooking, Eldergames, Retirement Party, VAP-M, Watermons, GAME	11
**Knowledge tests**<br>The game includes tests designed to probe knowledge learned throughout training	MACBETH, VAP-M	2
**Leaderboard/high score**<br>The game includes comparison of players over time, and ranks them.	Xcog, The Great Brain Experiment, Eldergames, Visual Search	4
**Levels**<br>The game includes levels of differing design which the player must progress through in order to advance	Supermecha, Megabot, WMTrainer, Kitchen & Cooking, BAM-COG, Cogoland, Card-Pairing, GAME	8
**Lives**<br>The game includes a life mechanic whereby the player is punished for making mistakes by withholding play-time.	WMTrainer	1
**Minigames**<br>The game includes/is broken into several different games with substantially different mechanics	Playmancer, Xcog, Braingame Brian, Kitchen & Cooking, BAM-COG, The Great Brain Experiment, Wii Tests, Tap the Hedgehog, Eldergames, TENI, Cogoland	11
**Negative feedback**<br>Making mistakes in the game is punished by deduction in points, temporary loss of control, a message stating “WRONG!” or similar	Playmancer, Supermecha, Megabot, Neuroracer, WMTrainer, Kitchen & Cooking, BAM-COG, Shapebuilder, The Great Brain Experiment, TENI, EM-Ants, Ghost-Trap, Space Code, Space Matrix, Retirement Party, Whack-a-mole, Watermons, GAME	18
**Online option**<br>The game may be played online, away from the laboratory	MACBETH, BAM-COG, Shapebuilder, The Great Brain Experiment, EM-Ants, Ghost-Trap	6
**Positive feedback**<br>The game rewards good play with points, congratulating messages, encouragement, upgrades, new levels or similar.	Playmancer, Supermecha, Megabot, Xcog, Braingame Brian, Neuroracer, WMTrainer, ABMTApp, Kitchen & Cooking, Shapebuilder, The Great Brain Experiment, Tap the Hedgehog, Groundskeeper, Eldergames, TENI, EM-Ants,	24

	Ghost-Trap, Space Code, Space Matrix, Retirement Party, Visual Search, Whack-a-mole, Watermons, GAME	
**Task presented as puzzles**<br>The tasks within the game are presented as logic puzzles that must be solved for their own sake, as opposed to being embedded within a narrative structure.	BAM-COG, Wii Tests, Eldergames, TENI, VAP-M, Card-Pairing, GAME	7
**Tangible Interface**<br>The game is played using a novel tangible interface	Smart Harmony, Wii Tests, Tap the Hedgehog, Groundskeeper, Eldergames, Cogoland	6
**Score**<br>The game uses a scoring system to reward player's actions	Playmancer, Supermecha, Xcog, Braingame Brian, Neuroracer, WMTrainer, ABMTApp, Smart Harmony, BAM-COG, Shapebuilder, The Great Brain Experiment, Tap the Hedgehog, Eldergames, EM-Ants, Ghost-Trap, Space Code, Space Matrix, Visual Search, Whack-a-mole, Cogoland, Kitchen & Cooking, Card-Pairing, Watermons	23
**Score multipliers**<br>In addition to a scoring system the game uses 'streaks' or 'bonuses' to reward players for continuous high performance	Supermecha, Shapebuilder, Tap the Hedgehog, Eldergames, Space Code, Space Matrix, Visual Search	7
**Sound effects**<br>The game uses sound effects to enhance the play experience or reinforce action within the game world	Playmancer, Supermecha, Megabot, Xcog, Braingame Brian, Neuroracer, WMTrainer, ABMTApp, Smart Harmony, Kitchen & Cooking, BAM-COG, The Great Brain Experiment, Wii Tests, Tap the Hedgehog, Groundskeeper, Eldergames, TENI, EM-Ants, Ghost-Trap, Space Code, Space Matrix, Visual Search, Whack-a-mole	23
**3D Graphics**<br>The game uses a 3D engine	Playmancer, Braingame Brian, Neuroracer, Kitchen & Cooking, VAP-M, Cogoland	6
**2D Graphics**<br>The game is uses a 2D game engine. Faux-3D games, such as those that use isometry, are still classed as using 2D graphics	MACBETH, Supermecha, Megabot, WMTrainer, ABMTApp, Smart Harmony, Kitchen & Cooking, BAM-COG, Shapebuilder, The Great Brain Experiment, Wii Tests, Groundskeeper, Eldergames, TENI, EM-Ants, Ghost-Trap, Space Code, Space Matrix, Retirement Party, Visual Search, Whack-a-mole, Card-Pairing, Watermons, GAME	24

**Storyline**<br>The game hinges around a central storyline, however vague, which the player may work their way through. The storyline can be flexible, but there must be setting of the scene and an explanation for why the player must perform the task	MACBETH, Supermecha, Megabot, Xcog, Braingame Brian, EM-Ants, Ghost-Trap, Space Code, Space Matrix, Retirement Party, Watermons	11
**Strong Theme**<br>The game has a strong theme that links art, music or story within the game together. Theme may serve to give meaning to a task without a ‘reason’ for the task being explained	Playmancer, Supermecha, Megabot, Xcog, Braingame Brian, Neuroracer, WMTrainer, ABMTApp, Kitchen & Cooking, The Great Brain Experiment, Tap the Hedgehog, Groundskeeper, EM-Ants, Ghost-Trap, Space Code, Space Matrix, Whack-a-mole, VAP-M, Cogoland, Watermons, GAME	21
**Time pressure**<br>The game involves completing tasks within limited time or under the impression that time is limited.	Playmancer, Xcog, WMTrainer, ABMTApp, Smart Harmony, The Great Brain Experiment, Tap the Hedgehog, Groundskeeper, Eldergames, EM-Ants, Space Code, Space Matrix, Retirement Party, Visual Search, Whack-a-mole, VAP-M, Cogoland, Kitchen & Cooking, Watermons	19
**Difficulty Levels**<br>The game includes varying levels of difficulty which are either automatically or manually adjusted to the player’s skill level.	Megabot, Xcog, Kitchen & Cooking, Shapebuilder, Wii Tests, Cogoland, GAME	7

Supplementary Figure 1: Bar chart showing the use of different game mechanics in testing and training games

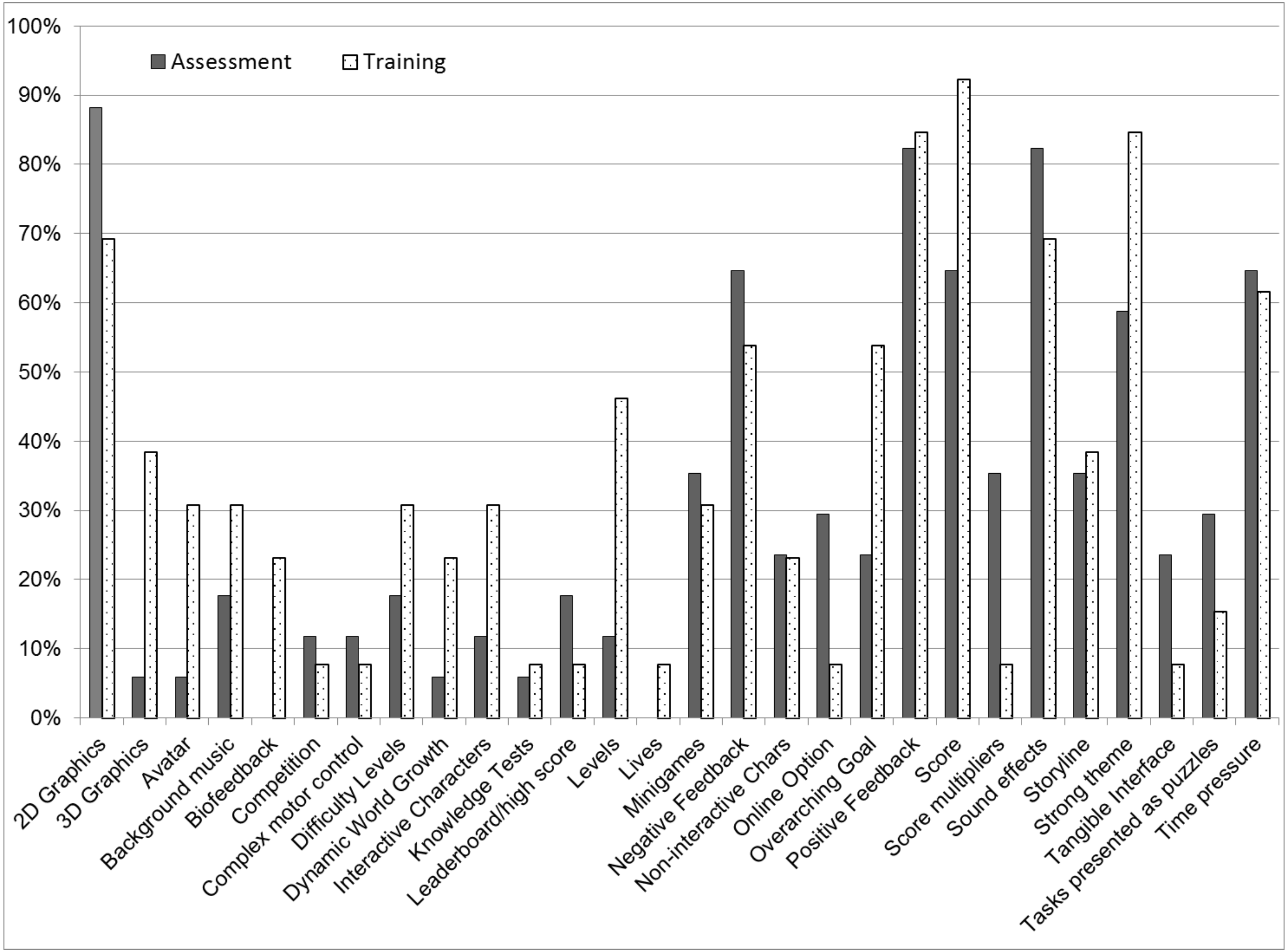

Supplement: Multimedia Appendix 2 [file games_v4i2e11_app2.pdf]
